# Naked barley: taxonomy, breeding, and prospects of utilization

**DOI:** 10.18699/VJGB-22-64

**Published:** 2022-10

**Authors:** K.A. Lukina, O.N. Kovaleva, I.G. Loskutov

**Affiliations:** Federal Research Center the N.I. Vavilov All-Russian Institute of Plant Genetic Resources (VIR), St. Petersburg, Russia; Federal Research Center the N.I. Vavilov All-Russian Institute of Plant Genetic Resources (VIR), St. Petersburg, Russia; Federal Research Center the N.I. Vavilov All-Russian Institute of Plant Genetic Resources (VIR), St. Petersburg, Russia

**Keywords:** naked barley, taxonomy, origin, genetics, grain quality, disease resistance, yield, breeding, голозерный ячмень, систематика, происхождение, генетика, качество зерна, устойчивость к болезням, урожайность, селекция

## Abstract

This review surveys the current state of taxonomy, origin, and utilization prospects for naked barley. The cultivated barley Hordeum vulgare L. incorporates the covered and naked barley groups. Naked barleys are divided into six-row naked barley (convar. сoeleste (L.) A. Trof.) and two-row naked barley (convar. nudum (L.) A. Trof.). The groups include botanical varieties differing in the structural features of spikes, awns, floret and spikelet glumes, and the color of kernels. The centers of morphogenesis for naked barley are scrutinized employing archeological and paleoethnobotanical data, and the diversity of its forms. Hypotheses on the centers of its origin are discussed using DNA marker data. The main areas of its cultivation are shown, along with possible reasons for such a predominating or exclusive distribution of naked barley in highland areas. Inheritance of nakedness and mechanisms of its manifestation are considered in the context of new data in genetics. The biochemical composition of barley grain in protein, some essential and nonessential amino acids, β-glucans, vitamins, and antioxidants is described. Naked barley is shown to be a valuable source of unique combinations of soluble and insoluble dietary fibers and polysaccharides. The parameters limiting wider distribution of naked barley over the world are emphasized, and breeding efforts that could mitigate them are proposed. Pathogen-resistant naked barley accessions are identified to serve as promising sources for increasing grain yield and quality. Main stages and trends of naked barley breeding are considered and the importance of the VIR global germplasm collection as the richest repository of genetic material for the development of breeding is shown.

## Abstract (ru)

В обзоре освещены вопросы современного состояния систематики, происхождения и перспектив использования голозерного ячменя. Представлены положения систематики культурного ячменя Hordeum vulgare L., в которой выделяются группы многорядного (сonvar. coeleste (L.) A. Trof.) и двурядного (сonvar. nudum (L.) A. Trof.) голозерного ячменя. Группы включают разновидности согласно особенностям строения колоса, остей, цветковых и колосковых чешуй и цвету зерновок. Отражены вопросы очагов формообразования голозерного ячменя, по данным археологии, палеоэтноботаники, разнообразию форм и сортов, а также рассмотрены вопросы происхождения голозерного ячменя по результатам ДНК-маркирования. Приведены основные зоны возделывания и возможные причины такого преимущественного или исключительного распространения голозерных ячменей в районах высокогорья. Обсуждается вопрос наследования признака голозерности ячменя и механизмов его проявления в соответствии с новыми данными в области генетики. Представлены характеристики биохимического состава зерна по белку, некоторым заменимым и незаменимым аминокислотам, β-глюканам, витаминам, антиоксидантам. Показано, что голозерный ячмень – ценный источник уникальных комбинаций растворимых и нерастворимых пищевых волокон и полисахаридов. Подчеркнуто значение параметров голозерного ячменя, которые ограничивают широкое распространение этой культуры в мире, и выделены направления селекционной работы для устранения этих недостатков. Приведены примеры образцов голозерного ячменя, устойчивых к вредным организмам, которые являются перспективными источниками для повышения урожайности и качества зерна, а также сохранения экологической чистоты и безопасности продукции. Отражены этапы и направления селекционных работ с голозерным ячменем и показано значение мировой коллекции ВИР как основного источника генетического материала для развития селекции.

## Introduction

Barley has been one of the most important cereal crops cultivated in all the world’s agricultural areas since ancient times. Barley belongs to the genus Hordeum of the Triticaceae family and is an obligate self-pollinator with a diploid set of chromosomes (2n = 14). It is rightfully recognized as a universal crop in terms of both the distribution scope and use. It is the fourth among the most important cereals in the world after wheat, maize and rice.

The wide area of distribution and a long cultivation history induced the rich intraspecific diversity of H. vulgare L. This cultivated species is divided into two subspecies: multi-row (H. vulgare L. subsp. vulgare) and two-row (H. vulgare L. subsp. distichon (L.) Koern.). They include groups of covered and naked botanical varieties. Among naked barleys, the groups of multi-row (сonvar. coeleste (L.) A. Trof.) and two-row naked barley (convar. nudum (L.) A. Trof.) were identified (Luk’yanova et al., 1990).

A specific feature of the naked barley groups is that their grains are bare and do not adhere to glumes, so kernels are easily separated from them when threshed. Naked barley is a valuable source for breeding for grain quality. Various forms of naked barley are characterized by a high content of protein and essential amino acids, primarily lysine, phenylalanine, methionine and threonine, fats, β-glucans, sterols, tocotrienols, flavonols and phytophenols possessing antioxidant activity (Aniskov et al., 2015; Meints et al., 2021).

Naked barley has its drawbacks. The main disadvantage of naked barley is the protrusion of the central radicle beyond the sphere of the grain surface, which leads to damage to the embryo during threshing. The crop is characterized by low adaptability to changing environmental conditions and low resistance to drought, lodging, and various diseases. It means that breeding work with naked barley should be aimed not only at increasing its positive properties but also at eliminating its major disadvantages. Currently, plant genetic resources are being actively analyzed in order to identify sources and donors for the main breeding trends.

## Taxonomy

The history of barley classification dates back to ancient times. In 1747, C. Linnaeus laid the foundations of scientific plant taxonomy, covering a huge botanical diversity, including barleys. The classification was based on the number of fertile spikelets in each joint of the spike and the density of the spike itself. According to the Hordeum L. classification, there were four cultivated barley species within the genus, and the botanical varieties of naked barley, var. nudum L. (two-row) and var. coeleste L. (six-row), were identified within those species, i. e., already at that time the division of barley into covered and naked forms existed (Bakhteev, 1955; Trofimovskaya, 1972).

A great contribution to the development and further evolvement of the intraspecific classifications of barleys was made by such scientists as C.B. von Trinius, J.C. Doll, C. Koch, R.E. Regel, S.A. Nevsky, N.I. Vavilov, A.A. Orlov and F.Kh. Bakhteev. The naked barley was identified as a separate subspecies by J.C. Doll. The classifications by A.A. Orlov and F.Kh. Bakhteev recognized naked and covered barleys as varieties within different species and subspecies (Orlov, 1936; Bakhteev, 1955).

The contemporary classification, used at the N.I. Vavilov All-Russian Institute of Plant Genetic Resources (VIR) and based on the works by N.I. Vavilov, R. Mansfeld, S.A. Nevsky, F.Kh. Bakhteev and A.A. Orlov, was presented by A.Ya. Trofimovskaya (Trofimovskaya, 1972). The cultivated species H. vulgare L. is divided into two subspecies: multi-row (H. vulgare L. subsp. vulgare) and two-row (H. vulgare L. subsp. distichon (L.) Koern.). They include the groups of covered and naked botanical varieties. Among naked barleys, the groups of multi-row naked barley (сonvar. coeleste (L.) A. Trof.) and two-row naked barley (convar. nudum (L.) A. Trof.) were identified (Trofimovskaya, 1972; Luk’yanova et al., 1990). Naked barley groups are characterized by a large number of endemic varieties from various foothill and highland areas. This classification mirrors the enormous polymorphism of species and botanical varieties within this genus, including naked barley.

The group of multi-row naked barley, H. vulgare L. subsp. vulgare convar. coeleste (L.) A. Trof., includes 58 botanical varieties (Luk’yanova et al., 1990). Its typical feature is that all three spikelets sitting in the grooves of the spike’s stem are fertile in most of the spike and have normally developed grains, while the grains themselves are bare, which means that they are easily separated from the glumes. Botanica varieties within the group are distinguished by the width of glumes, the presence, length and smoothness of awns, the color of the spike, the density of the spike, and also by the color of grains.

The density of the spike differentiates varieties into those with dense and lax spikes. Dense spikes are further subdivided into dense and very dense ones. A very dense spike is characteristic of the following botanical varieties: var. nudipyramidatum Koern., var. uljassutaicum Vav. et Orl., var. subnudipyramidatum Orl., var. micrurum Vav. et Orl., and var. latinudipyramidatum Vav. et Orl. These botanical varieties belong to the Japanese, Chinese and Mongolo-Tibetan agroecological groups. Their specific feature, in addition to a dense spike, is a stunted growth habit. Botanical varieties with a dense spike are: var. revelatum Koern., var. ancoberense Vav. et Orl., var. brevisetum Regel, var. nanum Vav. et Orl., and var. latirevelatum Vav. et Orl. These botanical varieties, belonging to the Japanese, Chinese and Abyssinian agroecological groups, are common in Japan, China, and Ethiopia.

Botanical varieties within the group of multi-row naked barley are described as having three-lobed appendages instead of awns (furcae): var. trifurcatum (Schlecht.) Wender., var. pseudotrifurcatum Langsd., and var. aethiops Koern., obtained from China, Mongolia and Ethiopia, but some botanical varieties with the same feature have been identified in hybridization nurseries. This group also includes botanical varieties with awns or furcae only on the middle spikelets; the lateral ones are awnless or have only small rudiments of awns: var. cornutum Schrad., var. cornutifor- me Aoberg., var. subaethiops Koern., var. nuditransiens Koern., and var. nudijaponicum Vav. et Orl., from Japan, South Africa, Tibet, and hybrids obtained in hybridization nurseries.

Grain color features are the criteria for differentiating many botanical varieties. Naked barley grains are of various colors: yellow (var. coeleste L. and var. brevisetum Regel), green (var. himalayense (Ritt.) Koern., var. urgaicum Vav. et Orl. and var. kobdicum Vav. et Orl.), purple (var. violaceum Koern., var. gobicum Vav. et Orl. and var. uhangaicum Vav. et Orl.), black (var. duplinigrum Koern. and var. aethiops Koern.), and brown (var. tibetanum Vav. et Orl.); there are also diverse shades of these colors acquired during splitting in crosses. Botanical varieties with yellow grains occur everywhere. Varieties with green grains are also widespread, but they predominantly belong to Asian agroecological groups. The purple grain color is observed in botanical varieties from the Mongolo-Tibetan and Chinese agroecological groups. Varieties with black grains belong to the Abyssinian agroecological group. The variety with brown grains belongs to the Tibetan agroecological group.

The two-row naked barley group, H. vulgare L. subsp. distichon (L.) Koern. convar. nudum (L.) A. Trof., is characterized by the fact that of the three spikelets sitting in the grooves of the spike’s stem only one spikelet, the middle one, is always fertile, with a normally developed grain; the grains are bare, i. e., they are not firmly adhered to the glumes, so they are easily separated from them during threshing. This group consists of 38 botanical varieties. Similarly to the multi-row naked barley group, the varieties within this group are differentiated according to the morphological features of the spike and the color of grains. All varieties mainly belong to the Abyssinian, Dagestani, Japanese and Indian agroecological groups; besides, many varieties were obtained in the process of crossing in hybridization nurseries. Botanical varieties with a dense spike (var. gymnocrithum Koern., var. neogenes Koern., var. nudimelanocrithum Giess., etc.) belong to the Abyssinian agroecological group.

Botanical varieties are differentiated within the group according to the presence/absence of awns, their length, and the presence of awn appendages (furcae). The varieties that have three-lobed appendages instead of awns (furcae) are var. nudifurcatum Regel, var. zhukovskii Chodk., var. sublaxum Koern., and var. gymnospermum Koern. The awnless varieties are var. dupliatrum Koern., var. duplialbum Koern., var. subduplialbum Koern., and var. subdupliatrum Koern. These varieties belong to Asian agroecological groups or resulted from crosses in hybridization nurseries.

The two-row group of naked barley contains varieties that are described to have lateral spikelets completely reduced and represented by only glumes: var. nudideficiens Koern., and var. daghestanicum Vav. et Orl. They belong to the Dagestani agroecological group.

Color is also a distinctive feature of many botanical varieties. Similarly to the multi-row naked barley, grains can be yellow (var. nudum L. and var. colonicum Orl.), green (var. viride Vav. et Orl., var. virideinerme Giess. et al. and var. daghestanicum Vav. et Orl.), purple (var. nudidubium Koern. and var. janthinum Koern.) or black (var. nigrinudum Vav. and var. nudimelanocrithum Giess. et al.), or manifest various other shades. The distribution of varieties with different grain colors is similar to that of the multi-row naked barley group.

The VIR global collection includes more than 1230 naked barley accessions collected from all over the world. These accessions are valuable genetic material for barley breeding and can serve as a source for the development of high-yielding naked barley cultivars adaptable to the environments of various regions of the Russian Federation.

## Origin and distribution of naked barleys

Very little is known about the origin of naked barley, because no targeted studies have been performed on the subject; it has been considered only in the context of the origin of barley in general. The timeframe of the origin of naked barley started to be discussed for the first time when multi-row naked and covered barleys were discovered during the excavations at Ali Kosh. A radioisotope analysis ascertained that naked barley appeared approximately in the period of 7900 B.C. These data indicate that naked barley evolved much later than covered barley which grew in the Pre-Pottery Neolithic (9700–9300 B.C.) (Helback, 1959). The very process of naked barley emergence also remains not quite clear. The most common hypothesis is the occurrence of a mutation in the gene that controls the process of husk formation in grains.

Scientists of VIR found out in their research that naked barley had three main foci of morphogenesis (Vavilov, 1965; Luk’yanova et al., 1990). N.I. Vavilov perceived these foci as “the loci of morphogenesis”, “extremely small spaces”, where wild plant species were domesticated by man. Such foci are identifiable by the data of archeology and paleoethnobotany, but they are mainly identified according to the modern varietal diversity of cultivated plant species and forms. It is important to understand that ancient foci could appear in different parts of the continents (i. e., polytopically) and at different times (heterochronously); later another phenomenon became known – repeated domestication (redomestication) of plant species against the background of a well-established ancient assortment.

The first focus is Southeast Asia and mountainous Central and Western China with the lowland areas adjacent to it. Naked barley is cultivated there mainly in mountainous areas at an altitude of at least 2000 m. The second focus is Northeast Africa (mountainous regions of Ethiopia), where naked barley varieties are represented by endemic forms. The third focus includes Western Asia: Turkey, Transcaucasia, Iran, and Tajikistan (Vavilov, 1957).

Recent efforts have been intensively associated with DNA markers. The data obtained can shift the concept of naked barley domestication to a higher level and confirm or refute the existing hypotheses of the origin and distribution of naked barleys.

One of the hypotheses concerning the origin of naked barley is monophyletic origin in the territory of Southwestern Iran, from where it started to migrate to other regions. This assumption is based on the analysis of the dominant SCARsKT7 marker, which is closely associated with the nud locus (Taketa et al., 2004). The highlands of the Himalayas can be considered as a possible center of domestication for naked barley (Badr et al., 2000) due to a number of distinctive features (Xifeng et al., 2013). Recent studies, however, manifest the opposite view. Based on the whole genome data and the published exon-swapping resequencing data for 437 accessions, the authors showed that the modern Tibetan barley (Hordeum vulgare L., qingke) was derived from domesticated oriental barley and introduced into Southern Tibet, presumably via Northern Pakistan, India, and Nepal, about 3500–4500 years ago. The scant genetic diversity of qingke suggests that Tibet can be ruled out as a center of origin or domestication of barley. The rapid decrease in genetic diversity on the way from domesticated oriental barley to qingke can be explained by the effect of isolation in the Tibet region from 2000 to 4500 years ago (Zeng et al., 2018).

In addition to the hypothesis on monophyletic origin of naked barley from Southwestern Iran, there is also a hypothesis about multiple independent origin of naked barley. It is based on a comparative morphological analysis of varieties from different foci of morphogenesis and consists in the independent emergence of naked barleys in several centers of crop origin (Helback, 1959).

The groups of multi-row and two-row naked barleys occur in all areas of barley cultivation. They are most widespread in Southeast Asia (China, Japan, and the Republic of Korea), Northeast Africa (Ethiopia and Eritrea), and Central Asia – in mountainous regions (Pamir, Tibet, Tajikistan, Mongolia and India) (Luk’yanova et al., 1990). The most common varieties of these groups are var. coeleste L., var. himalayense (Ritt.) Koern. and var. nudum L. Crop areas under naked barley in the abovementioned countries are not uniform: in some countries they reach 95 % of the total barley crop area, while in others only 50 % or less. The areas under naked barley abruptly diminish in the direction from east to west. In Russia, naked barley is cultivated on a small scale due to a number of factors limiting its distribution (Tetyannikov, Bome, 2020).

There are two viewpoints on the reasons for the spread of naked barley mainly in mountainous areas. Some authors attribute this phenomenon to the active use of naked barley for food needs (Helbaek, 1966; Nevo, 1992), while others hypothesize that naked barley is better adapted to such conditions (Harlan, 1979). The second point of view was proved by A.A. Pomortsev et al. (1996) who studied the dynamics in the genotypic composition of barley populations obtained from crosses between the cultivars Moskovsky 121 (tworow, covered) and Dzhau Kabutak (six-row, naked, var. himalayense). The hybrids were grown concurrently from F2 to F9 in the Pamirs (2600 m above sea level) and from F2 to F10 in Moscow. As a result, it was shown that under the impact of natural selection the dynamics of populations according to marker loci during reproduction in the Pamirs and in Moscow was different and led to the divergence of populations. Under the conditions of the Pamir highlands, the selection was targeted against plants with a covered grain and two-row spike, while in the environments of Moscow the selection was targeted against plants with naked grains, which confirms the second viewpoint: naked barley is more adapted to growing in highland environments than covered barley (Pomortsev et al., 1996).

## The gene controlling grain nakedness and the mechanism of its action

It is currently recognized that the difference between covered and naked barleys is controlled by a single locus. Grain covering is classified as a dominant trait, and nakedness as a recessive one. The genetic locus was assigned to the long arm of barley chromosome 7H and named nud (from the word nudum) (Gerasimova et al., 2020). The nud gene is located at a distance of 0.3 cM from the proximal end and 1.2 cM from the distal one in the region (SCAR) of KT 2 and KT 4 (Kikuchi et al., 2003). The structural part of the nud gene consists of two exons and one intron.

The Nud gene is present in covered barley; it encodes the transcription of the ethylene response factor (ERF) family, which belongs to the group of Wax Inducer 1/Shine 1 (WIN1/SHN1)-like transcription factors. The said factor controls lipid biosynthesis and encodes protein of 227 amino acids (Taketa et al., 2008). There are three allele variations at the nud locus, designated as nud 1.a, nud 1.b, and nud 1.c. The nud 1.a allele is the result of a Nud deletion. The nud 1.b allele contains a nucleotide substitution of thymine with adenine in the second exon, which leads to a substitution of valine with aspartic acid at position 134. The nud 1.c allele has a 1 bp deletion in the second exon, which causes a reading frameshift and generates a premature stop codon, resulting in a truncated protein sequence of 199 amino acids.

The mechanism of forming hullness or nakedness on barley grains is not yet fully understood. The version most often encountered in publications is that the recessive nud gene is in an intact state and does not generate an adhesive lipid layer between the epidermis of the grain pericarp and the glumes, which allows them to be freely separated during threshing. As for the dominant Nud allele, it controls the biosynthesis of lipids which contribute to the adhesion of the glumes to the caryopsis and the formation of covered barleys (Taketa et al., 2008; Hoad et al., 2016).

Recent works on the nud locus sequencing showed that in all naked barley accessions this gene was characterized by a 17-kb deletion or the presence of the non-synonymous SNP T643A when compared with the functional Nud gene (Yu et al., 2016). The analysis of X-ray-induced naked grain mutants confirmed that the Nud gene carried non-synonymous single-nucleotide polymorphisms in all cases. It was also demonstrated that site-directed mutagenesis of the Nud gene induced the appearance of naked grains on initial transgenic plants (Gasparis et al., 2018). In addition to the normal Nud deletion mutation contributing to hullness, a new nud allele, designated nud 1.g, was identified in three naked barley varieties collected in Tibet. The nud 1.g allele contains the non-synonymous T643A SNP, unlike the functional Nud gene. Genetic analysis showed that SNP T643A nud 1.g co-segregates with the naked phenotype. Besides, the in silico prediction of functionally conservative sites and three-dimensional structures showed that the amino acid substitution (valine with aspartate) induced by SNP T643A could lead to a dramatic structural change in Nud that could result in the loss of its function. This study provides evidence of a possible new mechanism underpinning the origin of the naked phenotype of domesticated barley in Tibet (Yu et al., 2016).

More and more research is focused on the work with naked barley. In 2020, an article was published where the authors demonstrated a targeted change in the first exon of the Nud gene with the help of the RNA-guided endonuclease Cas9, which led to the conversion of the phenotypic features of grain hullness into nakedness. The covered barley cultivar Golden Promise served as the target research material, while the changes were implemented through the mediated DNA transfer by agrobacteria (Gerasimova et al., 2020).

However, a limited number of studies on this topic and broad variability of the source material that has not yet been fully studied impede the efforts to throw light on the molecular mechanisms that regulate grain nakedness or hullness in barley grains. Perhaps, using a wider diversity of naked barleys from around the world and applying modern methods would help to identify new loci responsible for grain nakedness.

## Chemical composition of naked barley grains

The main feature differentiating naked barley from covered barley is the biochemical composition of its grain. Barley grain contains unique combinations of soluble and insoluble dietary fibers and polysaccharides along with lowmolecular- weight bioactive components (Madakemohekar et al., 2018). Naked barley exceeds covered barley in the content of nutrients, such as protein, some essential and nonessential amino acids, β-glucans, vitamins, macro- and micronutrients, phenolic and flavonoid compounds. It has been established that all useful components of barley grain are preserved during its processing, including such active antioxidants as proanthocyanidins (Zheleznov et al., 2013; Polonsky et al., 2021).

A physiologically important dietary component in naked barley grain is (1,3;1,4)-β-D-glucans. They help to reduce the risk of cardiovascular disease, maintain or reduce the amount of cholesterol in blood, decrease the risk of hyperglycemic syndrome, improve liver functions, and reduce overweight (Wirkijowska et al., 2012; Bozbulut, Sanlier, 2019). The dry matter of covered barley grain contains 4–8 % of β-glucan, but for naked barley this index can reach 16 %. Its content in barley grain is determined by varietal characteristics and environmental factors (Huth et al., 2002). Naked barley is characterized by a high vitamin E content (Moreau et al., 2007) and is considered a good source of phenolic compounds, such as derivatives of cinnamic and benzoic acids, proanthocyanidins, flavonols, flavanones, and flavones (Shen et al., 2016; Ge et al., 2021), which manifest antioxidant, anti-inflammatory, and antiproliferative effects.

## Parameters limiting wider distribution of naked barley

One of the main factors limiting wider distribution of naked barley is its low yield compared with covered barley. This is largely due to its low field germination caused by the protrusion of the radicle beyond the grain. It affects the embryo’s resistance to the mechanical impact of threshing equipment, results in injuries, and leads to a decrease in field germination of its seeds.

In order to overcome this disadvantage of naked barley in breeding practice, it is necessary to control the morphology of the grain shape and the nature of the embryo’s positioning (Tetyannikov, Bome, 2020). Quite a few lines with oval-shaped and even rounded grains have already been developed by crossing the Canadian naked barley cultivars McGwire and BRL-6 with the covered cultivars Getman, Vakula, Linus and others (Kirdoglo et al., 2013). A very promising naked barley accession, 95683/73 (k-27730) from Germany, was identified in the VIR collection. Its grain is shortened (7.2 mm) but rather wide (3.8 mm) and maximally thick (2.8 mm). Such parameters make this accession uniquely important for practical use in naked barley breeding. Besides, accessions with the optimal grain shape were identified: Alar-Erd-Ene from Mongolia, Hora from the Netherlands, 1218-524 from the Czech Republic, and S-257 from Mexico (Malashkina, 2008).

Long ago it was shown that covered barley cultivars were more productive than naked ones. However, many authors who studied naked barley observed that the glumes, tightly adhered to the caryopsis, accounted for at least 12–14 % of the total bulk of covered barley harvest. Barley glumes themselves are the same straw, so it should be taken into account when measuring the real yield of covered barley (Gryaznov, 2014).

Barley yields are highly variable depending on environmental conditions, especially in arid areas (Gryaznov, 2014). Naked barley yields also vary significantly depending on the characteristics of cultivars. The productivity of naked barley forms was studied in many regions of Russia: in the Tyumen (Tetyannikov, Bome, 2020), Omsk (Aniskov et al., 2015) and Kemerovo (Zaushintsena et al., 2007) regions and in the provinces of North Caucasus (Doroshenko et al., 2019), and others. Accessions with the highest yield and best adaptive properties were identified in those regions.

Besides, one of the most important agronomic traits of naked barley is its resistance to lodging in different environments. T.M. Bogdanova et al. (2001) identified single accessions resistant to lodging under the conditions of Northwestern Russia: KM 280 (k-29419, var. nudum, Czech Republic), Nacta (k-20928, var. nudum, Germany), and complex hybrids from Mexico (k-28019, var. nudum, and k-28083, var. neogenes). Later, under the same conditions, accessions resistant to lodging were selected from two-row barleys: k-29863 (var. neogenes, Czechoslovakia) and k-28083 (var. neogenes, Mexico), and from multi-row barleys: k-28961 (var. coeleste, India), k-4365 (var. coeleste, Belarus), and k-21319 (var. subnudupyramidatum, Japan) (Tyaglyi, 2007). Testing under the conditions of Tyumen Province revealed resistance to lodging scoring 9 points in the two-row accessions: k-22308 (H 2198 Ubamer Baco), k-23450 (H 2866 Coll. Halle EP 80), k-25008 (Local), and k-25855 (Ra 6), and in multi-row ones: k-30663 (C.I.11073) and k-30624 (C.I.10975) (Tetyannikov, Bome, 2020).

An important criterion for increasing the yield of naked barley in the areas of risky farming is its earliness. For example, when studying the earliness of barleys from the countries of Southeast Asia, two ultra-early forms from China were identified: k-15881 (var. coeleste L.) and k-15882 (var. nudipyramidatum Koern.), with an interval of 30–33 days between the germination and ear emergence phases (Zveinek, Kovaleva, 2017), which makes these accessions promising for cultivation in areas with unfavorable abiotic factors. Besides, a study of the extensive material from the VIR collection resulted in the identification of 16 early-ripening accessions, such as k-25090 (Mexico, var. nudum) and k-29820 (Ethiopia, var. nigrinudum) from the two-row group, and k-5489 (Ukraine, var. glabriduplinigrum) and k-24817 (Ethiopia, var. tibetanum) from the multi-row one (Bogdanova et al., 2001).

Practical cultivation of naked barley cultivars, including cv. Nudum 95, attests to the need for the development of measures to adapt such cultivars to local environmental conditions, which will increase their yield to the level of covered barley. Such technologies have already started to appear, but only for certain regions (Gryaznov, 2016; Gladkikh et al., 2019).

## Resistance to various diseases

Disease resistance of cultivars is one of the important reserves for increasing the yield and quality of grain as well as for maintaining the ecological cleanliness and safety of products.

Diseases caused by fungi, bacteria and viruses, and pests of barley occur each year in various regions of Russia, resulting in abrupt decreases in grain yield and quality. They affect the normal rhythm of plant development, produce a negative impact on grain formation, reducing grain size and filling, and disrupt plant stands. Therefore, breeding for quality is closely associated with breeding for resistance to diseases and pests. The most profitable and safest way to reduce grain contamination is to develop genetically resistant cultivars. Breeding problems cannot be solved without a constant search for new sources and donors of resistance, because plant resistance genes lose their effectiveness in the process of emergence and accumulation of virulence mutations in pathogen populations (Luk’yanova et al., 1990).

It is known that barley can be infected with a wide range of pathogenic fungi, and many of them can persist in the grain. The genera Bipolaris, Pyrenophora, Phaeosphaeria, Alternaria, Ustilago, Puccinia, Blumeria and Fusarium are considered the most common fungi infecting barley grain worldwide (Chen et al., 2016). Reduction of losses in harvests and valuable grain qualities requires careful selection of source material and involvement of the most resistant sources and donors in breeding practice to develop new cultivars.

Fusarium is a common disease of cereals, such as wheat, maize or barley, and can lead to an abrupt decrease in yield and product quality (Polisenska et al., 2020) through the formation of mycotoxins. Mycotoxins in the human organism cause anorexia, vomiting, diarrhea and, in high doses, intestinal bleeding; sometimes they produce additional effects, such as impaired immune function. In plants, mycotoxins inhibit protein synthesis, while fungal enzymes lead to protein degradation, thus inducing plant defense mechanisms (Martin et al., 2018).

Plant resistance to Fusarium and accumulation of mycotoxins is a complex mechanism. Five major resistance classes have been established for wheat, barley and maize. Resistance of type I acts against initial penetration and infection of plants. Type II restricts the spreading of infection within the plant. Resistance of type III deals with grain infestation, type IV is associated with resistance and the ability to maintain yield, and type V combines all mechanisms of resistance to mycotoxin accumulation (Martin et al., 2018). Resistance of type V is suggested to be divided into two components. The first, called type V-1, is the resistance to toxin accumulation driven by metabolic transformation, including enzyme-catalyzed biochemical modification. The second component (type V-2) corresponds to the resistance obtained through inhibition of mycotoxin biosynthesis by endogenous compounds within the plant itself (Martin et al., 2017).

There is proof that a large number of various plant metabolites play a decisive role in the resistance to Fusarium: phenolic acids, flavonoids, carotenoids, tocopherols, benzoxazinoids, fatty acids, amino acids and their derivatives, carbohydrates, amines and polyamines, terpenoids, etc. (Gauthier et al., 2015; Atanasova-Penichon et al., 2016). They suppress reactive oxygen species, scavenge free radicals during lipid peroxidation and contribute to the establishment of a physical barrier against pathogenic infection, while some metabolites can interfere with the biosynthesis of mycotoxins (Siranidou et al., 2002). High β-glucan content in grain was also shown to contribute to the resistance of type V (Martin et al., 2018).

When studying covered and naked barleys, some researchers demonstrated that covered barley turned out to be more resistant (Warzecha et al., 2010), while others found very low content of toxins in naked barley cultivars, justifying this by the fact that a significant amount of toxins remains in glumes (Buerstmayr et al., 2004). The latest data on the specific features of barley resistance to Fusarium witness that the advantage of some naked barley forms over covered ones in terms of the content of metabolite groups that enhance the resistance of type V should be taken into account.

A study of Fusarium resistance in barley cultivars, conducted by domestic researchers, identified 14 highly resistant accessions. Five of them were naked forms (k-2946, k-11070, k-11073, k-11076 and k-11082) with large grains, but prone to lodging and susceptible to powdery mildew (Gagkaeva, Gavrilova, 2009).

Powdery mildew (causative agent: Blumeria graminis (DC.) Golovinex Speerf. sp. hordei Marchal), brown rust (Pucciniahordei G.H. Otth.) and Helminthosporium are among the most widespread and harmful diseases of barley in Russia (Kusch, Panstruga, 2017). Long-term resistance to the powdery mildew pathogen of barley cultivars is provided almost all over the world by the mlo11 gene and, to some extent, mlo9 (Radchenko et al., 2020). A number of naked barley accessions with resistance or low susceptibility to powdery mildew were identified: Dublet (Belarus), Omsky Golozerny 1 (Russia), k-26648 (Pakistan), Buck CDC, CDC VC Gwire and CDC Dawn (Canada), k-3038 (Turkmenistan), Orgeniepetite (France), NB-OWA (Nepal), etc. Resistance to leaf spots caused by Helminthosporium was observed in Buck CDC and Bowman (Canada), 84469/70, k-3038 (Turkmenistan), Dublet (Belarus), Brunee (Ethiopia), Orgeniepetite (France) and others. Complex resistance to both pathogens was demonstrated by the accessions: Dublet, Omsky Golozerny 1, Omsky Golozerny 2, Yudinsky 1, k-26648, 84469/70, Orgeniepetite, CDC Dawn, NB-OWA, k-3038, CDC VC Gwire and E.E.B.N.46. They are recommended for use in breeding programs targeted at resistance to fungal diseases (Doroshenko E.S., Doroshenko Ed.S., 2018).

Accessions k-5448, k-8682 and k-17554 with powdery mildew resistance were identified while testing Ethiopian accessions at Pushkin and Pavlovsk Laboratories of VIR as potential sources of the mlo11 gene allele in the development of cultivars resistant to powdery mildew. Accession k-5448 (Abyn 8, var. duplinigrum, Ethiopia) was also resistant to net blotch: the plant damage did not exceed the score of 1 point (Alpateva et al., 2016). A stock of accessions resistant to fungi was obtained during the study of naked barley cultivars from the VIR collection. The best accessions that retain powdery mildew resistance for 30 years are k-2930 (var. violaceum, China), k-5983 (var. coeleste, Afghanistan) and k-3282 (var. nigrinudum, Ethiopia) (Bogdanova et al., 2001).

In addition, wider distribution of naked barley is hampered by its susceptibility to smut fungi. Among numerous pathogens of cereals, smut fungi manifest one of the highest levels of harm due to the fact that they are ubiquitous, cause a very significant decrease in yield and worsen the quality of grain. They can also provoke reduction of dry matter accumulation in grain, shortening of the ear length, and a decrease in tillering and the number of grains per ear (Bechtol’d, Orlova, 2018). Phytopathological analysis of 40 naked barley accessions for covered smut showed that only three cultivars had absolute resistance (0.0 %) to the pathogen: Chugokuhadaka N2 (Japan), Buck CDC (Canada), and k-30313 (Ethiopia).

Mid-ripening naked barley accessions were found to be more affected by smut fungi compared to mid-late and early ones. Seeds infected with loose smut have a lower absolute weight (by 10–20 %) and their field germination deteriorates (Zhichkina, Stolpivskaya, 2015). As a result of the studies (2005–2007), 8 covered and 4 naked cultivars were identified for being not affected by false loose smut and covered smut, including the references Omsky 85 and Omsky Golozerny 2. Accessions combining resistance to false loose, covered and loose smuts were identified. They were recommended to breeders for inclusion into crosses in order to obtain immune cultivars (Meshkova, Sabaeva, 2009). Accessions k-23851 (var. himalayense, Mountainous Badakhshan) and k-21544 (var. trifurcatum, Bolivia) from the VIR collection were resistant to loose smut for 12 years (Bogdanova et al., 2001). They are also of interest as promising sources.

## Breeding improvement of naked barley

Historically, the study of naked barley was started by such scientists as N.I. Vavilov, A.A. Orlov and F.Kh. Bakhteev. They drew the attention of plant breeders, geneticists and agronomists to the diversity of naked barley forms available in the global collection. They identified distinctive features and areas of distribution of these forms, assessed their agroecological characteristics, described the scope of their application, and also initiated the collecting of naked barely samples from all over the world (Khod’kov, 1985).

In Russia, the areas where naked barley was grown were very limited. The first information about naked barley cultivation in Eastern Siberia dates back to the beginning of the 19th century. It was mostly in those years that the so-called Himalayan barley appeared among cultivated barleys. This form was borrowed in 1826 by S.I. Gagarin, Vice-President of the Imperial Moscow Agricultural Society, from Archduke John of Austria (Surin, 2011). According to L.E. Khod’kov (1985), in the early period of domestic breeding, only some agricultural institutions were interested in naked barley. For example, Himalayan naked barley was studied for several years at the Zapolskaya Experiment Station in the late 19th century. In 1914, the first cultivar of naked barley, Nudum 155, was released at the Dnepropetrovsk Experiment Station. It was obtained through individual selection.

However, this is not the first information about the cultivation of naked barley in the Russian Federation. An analysis of fossil plant remains discovered by the expedition of the Dagestan Branch of the USSR Academy of Sciences during excavations of ancient settlements near the village of Gilyar in Southern Dagestan showed that 4.5–5 thousand years ago (in the Ancient Bronze Age) local residents cultivated wheat and barley. With this in view, it is noteworthy that naked barley was the most widespread (Omarov, 1981). Local highlanders even singled out naked barley as an independent crop, different from ordinary cultivated barley. Naked barley was exclusively cultivated in the mountainous and alpine areas of Dagestan, where its grain was used for food purposes.

Naked barley appeared in the global collection of VIR at the very beginning of its establishment. It is interesting that the first (k-1) registered number in the VIR catalogue was given to a sample of naked barley (var. himalayense) from Uzbekistan acquired in 1897. The first accessions of naked barley arrived to the VIR collection from completely different parts of the world: Uzbekistan, China, Ukraine, Armenia, Georgia, Germany, Romania, Kazakhstan, Latvia, France, Kyrgyzstan, etc., and from all over Russia: Saratov, Yaroslavl, Tobolsk and Vyatka Provinces, Dagestan, Kuban, Kursk Province, Don and Black Sea regions, Yenisey and Terek basins, etc., which proves the widespread growth of naked barley in Russia.

The State Variety Network has been testing naked barley almost regularly since 1927, while the research into this crop has become systematic. In the 1920–1930s, the naked barley cultivars Nudum Rostovsky 0289, Nudum Rostovsky 3001, Coeleste 086, Nudum 021, Coeleste 08, Byloe, Kolkhozny 7 and Nudum 92 were developed. However, for various reasons, almost all of them were not officially commercialized and zoned. Post-war breeding efforts also did not achieve outstanding results in the development of new naked barley forms. Thus, since the end of the last century, the attention of both individual plant breeders and various domestic breeding institutions has been drawn to the problem of the development of naked barley cultivars and their introduction into agricultural production, but almost all of them were unsuccessful. L.E. Khod’kov (1985) in his publication “Naked and Awnless Barley” (1985) analyzed the experience in the breeding work with naked barleys in the country and showed a number of promising improved forms of his own breeding.

Targeted research on the development of naked barley cultivars is currently being carried out in Canada, Japan, the USA, Sweden and the Czech Republic. Russia, Ukraine and Belarus are also interested in this subject. In Canada, at the end of the 20th century, such cultivars as Scout and Tupper (1980), Condor (1988), Buck and Richard (1990) were released; they currently occupy the area of more than 350,000 hectares (Aniskov et al., 2015). Besides, in 1997, the “waxy” barley was first developed in Canada; it exceeded common barley in the content of β-glucans by 32–41 %. In Belarus, the first studies on the development of naked barley cultivars date back to the 1970s. Those efforts resulted in releasing such cultivars as Golozerny 76, Belorussky 76, Golozerny 94 and Dublet (Trofimovskaya, 1972). Significant progress has been made in Switzerland, where several naked barley cultivars were included in the official catalogue in the late 1980s.

In the Russian Federation, breeding works with naked barley are actively carried out in the Siberian Research Institute of Agriculture, Krasnoyarsk Research Institute of Agriculture, Siberian Research Institute of Plant Production and Breeding, and Kemerovo Research Institute of Agriculture. At present, there are already six cultivars of naked barley listed in the State Register for Selection Achievements: Omsky Golozerny 1 (2004), Omsky Golozerny 2 (2008) and Omsky Golozerny 4 (2020), Oskar (2007), Nudum 95 (2010), and Ergeninsky Golozerny (2020) (State Register…, 2021). However, these cultivars are adapted to the environments of certain regions, and in other regions their quantitative and qualitative traits are not manifested. For example, cv. Ergeninsky Golozerny is adapted to arid areas in the black earth zone, which makes it in demand in the south of Russia and other arid regions (Characteristics of Plant Varieties…, 2020).

Contemporary plant breeding has changed significantly. These changes are associated with the development of molecular marker technologies and the possibilities of sequencing (Khlestkina, 2013). They make selection by genotype possible, which significantly accelerates the breeding process (Jaganathan et al., 2020). Currently, SNP markers are widely used for genotyping (Agarwal et al., 2008; Jaganathan et al., 2020), and they are applied for both covered and naked barleys

The use of NGS (next-generation sequencing) technologies for studying naked barley is not as widespread as for covered barley. J. Hernandez et al. (2020) associated it with the problem of the absence of model naked barley. X. Chen (2014) studied two local naked barley cultivars XQ754 and Nimubai from Tibet using paired-end RNA sequencing on the Illumina HiSeq 2000 platform and derived their transcriptomes. All in all, 13.1 and 12.9 million 90 bp paired reads were produced from two cultivars. Based on databases, a description of the genes and conservative protein domains in the developing grain of naked barley was presented. Moreover, the sequences and expression levels of the genes associated with coding storage proteins and enzymes for starch synthesis and β-glucans were analyzed. Their temporal and spatial patterns were derived from the transcriptome data of the covered barley cultivar Morex (Chen et al., 2014). These data ensure the genetic potential to improve the qualitative traits of naked barley in future studies.

At present, the results of sequencing jointly with highthroughput genotyping technologies can be used for effective targeted selection of the desired genotypes among breeding lines, which will significantly accelerate the development of new barley cultivars with desired characteristics (Rozanova, Khlestkina, 2020).

The global barley collection held by VIR contains an extensive gene pool of naked barley. The group of multirow naked barleys constitutes a small part of the collection compared to covered ones and consists of 827 accessions, including 34 varieties. The two-row naked barley group includes only 303 accessions and 21 varieties. Many naked barley varieties in the VIR collection are endemic, very rare, and represented in the collection by single accessions, which makes the VIR collection a unique source of valuable genetic material.

## Economic importance of barley

Currently, the trend of food production from various cereals meeting the dietary needs of humans is actively developing. The dietetic, preventive and therapeutic effect of such food products on a human organism is based on the biochemical composition of cereal grains. In recent years, breeding practice has been aimed at developing high-yielding cultivars combining the maximum content of biochemical components and their optimal ratio with other grain quality indicators and resistance to various abiotic and biotic stressors (Loskutov, Khlestkina, 2021). Barley is among such crops: it is an important source of food and feed and a valuable industrial crop in many countries of the world. Barley demonstrates stable grain harvests every year: in 2020, according to FAO, the barley grain harvest amounted to over 151 million tons (http://www.fao.org/faostat/en/#data/QC, accessed June 25, 2021). About 75 % of the world’s barley production is used for animal feed, 20 % goes to the production of malt for the brewing industry, and only 5 % for food production (Blake et al., 2011).

## Use of barley for food purposes

Barley is highly adaptable to high-altitude climate conditions, drought, and soil salinity, which makes it an important staple food crop in North Africa and the Tibetan Plateau in China where other crops, such as wheat and rice, cannot produce high and stable yields (Moza, Gujral, 2016).

The list of food products from naked barley grain is currently expanding. Barley grain is used to make barley and pearl barley groats. Since the grain of naked barley is easily separated from glumes, the yield of groats from naked barley is higher than from covered barley. Naked barley cultivars meet all requirements for producing high-quality groats. Therefore, it seems advisable to replace a number of covered cultivars with naked ones for the production of groats (Borisonik, 1971).

Naked barley grain is also used to make flour. In highland areas, the flour from naked barley is mainly consumed as satu (flour made from roasted barley grains) and also mixed with flour made from other crops, such as wheat, buckwheat, millet and pea, to cook flatbread, dumplings and thukpa (Tibetan noodle soup). In addition to flour, a special traditional fermented drink (Chhyang) and distilled liquor are prepared from the grain for various cultural and religious events.

In Canada, products made from mixtures of wheat and barley flour in varying proportions are quite common. To preserve the biological value of the grain, whole grain flour is used, without siftings and technological waste (Trofimovskaya, 1972). In Italy, naked barley is widely used for processing into dietary barley flour or such products as quick breakfasts and coffee substitutes. To obtain barley coffee, the grain is roasted to a dark brown color and finely ground, and the resulting powdery mass is used as a coffee substitute.

The work is underway to use whole-grain oat and barley for the preparation of functional drinks, including plantbased milk. Such drinks are rich in B-group vitamins, complex carbohydrates, and various mineral components. Whole grains used in beverages also contain a wide variety of phenolic compounds with antioxidant activity (Fernandes et al., 2018/19).

In Russia, naked barley flour mixed with bread wheat flour is becoming more and more popular for baking purposes in order to enrich products with compounds useful for the human organism and significantly increase the nutritional and consumer value of bakery products.

A large number of experiments helped to identify the optimal ratio of wheat/barley flour (90/10 %) for bread. With such ratio, the characteristics of bread (organoleptic assessment of the surface and color of the crust, porosity and elasticity) are not inferior to those of the products made from pure wheat flour; however, it should be noted that an increase in the share of barley flour for bakery products to 25 % or more worsens the quality of products (Letyago, Belkina, 2019). Research is also underway to obtain bread products with increased antioxidant activity. Similar optimal ratios have been calculated for the addition of flour from the grain of the naked pigmented barley cultivar Granal 32, which shows increased antioxidant activity of 10 % (Gryaznov et al., 2019; Martínez-Subirà et al., 2020).

Including naked barley, rich in β-glucans and anthocyanins, in a balanced diet provides many health benefits, and such low-glycemic and fiber-rich dietetic foods may help to regulate blood glucose levels in healthy individuals and diabetic patients (Shakib, Gabrial, 2010; Martínez-Subirà et al., 2020).

## Use of barley for food purposes

Most of the world’s barley production (> 70 %) is used to meet the needs of animal husbandry. Barley as a fodder crop is widely utilized in Russia, CIS countries, Eastern Europe and Canada, where one of the most popular fodder crops, maize, is not so widespread (Avdeichik et al., 2009).

The grain of naked barley is a valuable high-energy feed, rich in protein and a number of essential amino acids, while the content of cellulose is low. Numerous studies on naked barley are aimed at including it in feeds with various enzyme supplements to improve the quality of animals and reduce rearing costs. Active introduction of naked barley grain into animal rations showed a positive effect mainly on laying hens (Dadashko et al., 2010), pigs (Tatarkina, 2019), broiler chickens (Teimouri et al., 2018), and geese (Toropova, Sukhanova, 2013).

## Other uses of barley

Actively developed is the trend when not only covered but also naked barley is used for the production of malt for the brewing industry and the sector dealing with other alcoholic drinks. Aqueous extracts from barley malt are also used in medicine, textile and leather industries. The possibility of using naked barley as a crop for brewing has been discussed for a very long time, but it is necessary to make adjustments to the technological process, for example by replacing natural filtration through films with artificial filters (Borisonik, 1971).

There is also a trend to use naked barley to make diastatic malt, which has a composition and enzymatic activity comparable to that of brewing and distilled malts, but superior to that of wheat malt brewed under identical conditions. The first advantage of such malt is a shorter maturation time than that of malting barley or wheat. Another advantage of naked barley malt is that it can be used directly for food purposes without the need to prepare malt extracts and syrups, as is the case with brewing and distillation industries (Bhatty, 1996). Besides, naked barley can serve as basic material for the production of fuel alcohol (Ingledew et al., 1995).

The latest studies on naked barley have shown its versatility for uses both for feed and food as well as for various production needs. Although researches have formed an opinion based on their long-term experience that there are difficulties in their approaches to promoting the use of naked barley, many of them believe in the expediency of at least partial replacement of covered barley with the naked one in order to increase produce quality and possibly reduce production costs.

## Conclusion

After a detailed review of published sources on naked barley, we can conclude that such forms of barley undoubtedly have a number of advantages over the traditional covered ones, such as easy separation of glumes during threshing, a more balanced biochemical composition, an increased content of protein, various amino acids, β-glucans and compounds with antioxidant activity, and a lower cellulose content, which increases its value as a fodder crop.

The VIR collection preserves and maintains more than 1230 naked barley accessions collected from all over the world. It can serve as a source for the development of highyielding cultivars of naked barley with adaptive properties, thus providing an advantage in producing high-quality harvests, while easy separation of the kernel from glumes would facilitate grain processing operations.

## Conflict of interest

The authors declare no conflict of interest.
